# Preoperative virtual simulation for synchronous multiple primary lung cancers using three-dimensional computed tomography lung reconstruction: a case report

**DOI:** 10.1186/s13019-020-01387-6

**Published:** 2021-01-07

**Authors:** Weichun Wu, Yimin Wu, Gang Shen, Guofei Zhang

**Affiliations:** 1 Department of Cardiothoracic Surgery, First People’s Hospital of Yuhang District; Yuhang Branch of the Second Affiliated Hospital of the Zhejiang University School of Medicine, 369 Yingbin Road, Hangzhou, 311100 China; 2 Department of Thoracic Surgery, The Second Affiliated Hospital of the Zhejiang University School of Medicine, 88 Jiefang Road, Hangzhou, 310009 China

**Keywords:** Preoperative simulation, Synchronous multiple primary lung cancers, Three-dimensional computed tomography reconstruction

## Abstract

**Background:**

As the positions and sizes of nodules in synchronous multiple primary lung cancer (SMPLC) patients differ, the development of surgical strategies to maximize long-term survival and preserved postoperative pulmonary function in SMPLC patients for whom surgical resection is an alternative strategy presents challenges.

**Case presentation:**

We provide a case managed through video-assisted thoracoscopic surgery (VATS) resection using three-dimensional computed tomography lung reconstruction (3D-CTLR) to reconstruct lobes containing pulmonary nodules to preoperatively simulate and intraoperatively guide the extent and method of resection.

**Conclusion:**

The successful attempt demonstrates a technically simplified, feasible alternative to preoperative plans utilizing less invasive VATS to manage SMPLC.

## Background

Although there is no consensus as to diagnostic and therapeutic methods in synchronous multiple primary lung cancer (SMPLC) cases, surgical resection, especially with the mini-invasive technique of video-assisted thoracoscopic surgery (VATS), has been accepted as an alternative strategy for certain patients in this group [1]. However, controversies related to the method of resection still exist. To obtain a prognosis similar to that of solitary primary lung cancers (SPLCs), even those of a similar histologic subtype, and preserve postoperative pulmonary function, different surgical procedures based on the experience of the surgeons; the patient’s age and cardiopulmonary function; and the size, quantity, and distribution of nodules have been individually adopted [2, 3]. Three-dimensional computed tomography lung reconstruction (3D-CTLR) can accurately determine the relative positions of these nodules in the lobes. Here, we provide a typical clinical example to demonstrate a procedure to reconstruct lung lobes containing pulmonary nodules using 3D-CTLR, which depicts the relative relationship between nodules and lobes to simulate and intraoperatively guide the extent and method of surgical resection.

## Case presentation

**Fig. 1:**
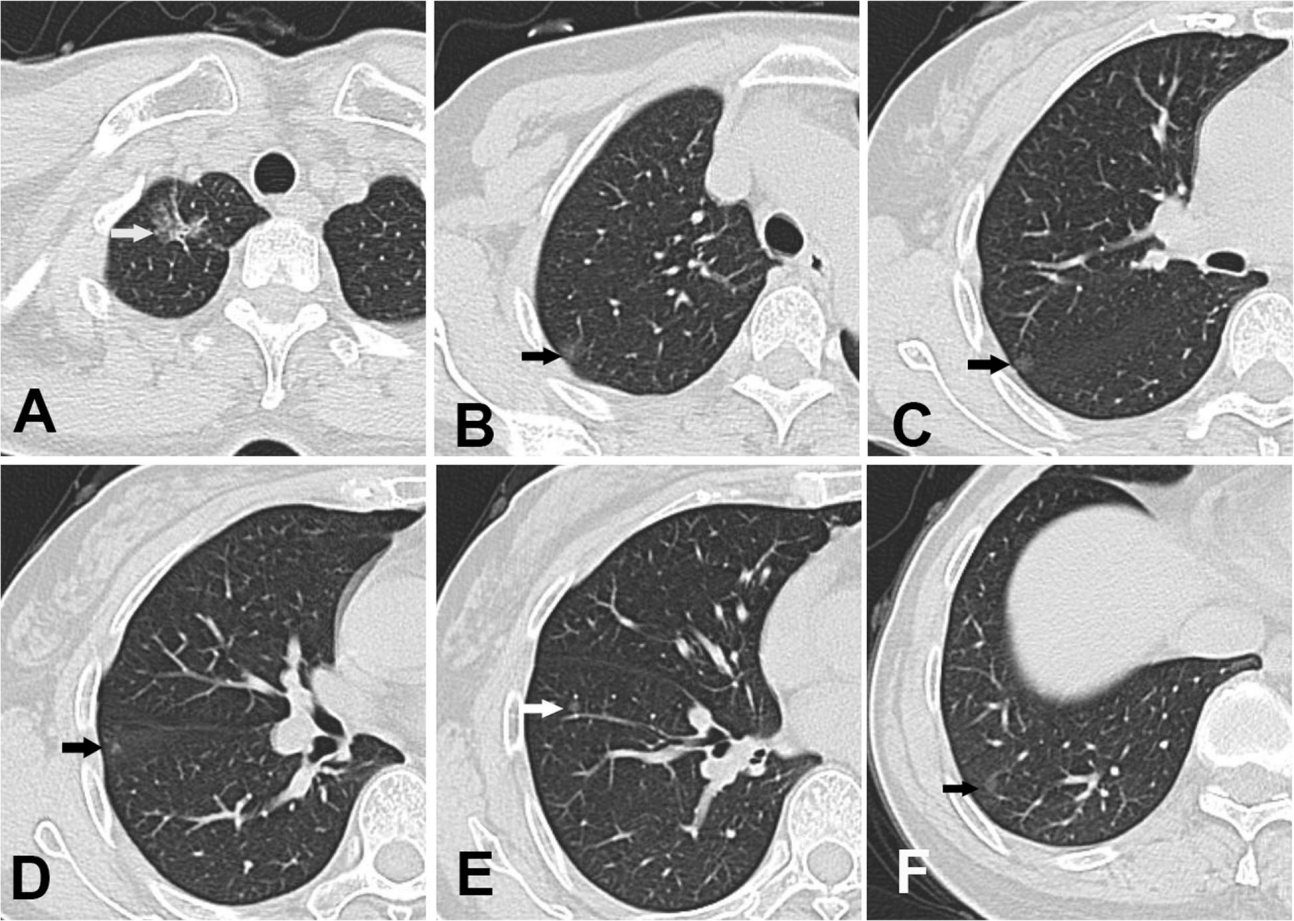
High-resolution computed tomography images of patient 1 reveal six ground-glass opacity (GGO) lesions in the right lobe (arrows). **a** One nodule in the apical segment of the right upper lobe. **b**-**d** Three nodules in the posterior segment of the right upper lobe. **e**, **f** Two lesions in the right lower lobe

A 58-year-old woman was admitted with eight ground-glass opacity (GGO) lesions in the bilateral lobes at preoperative HRCT (four in the right upper lobe, two in the right lower lobe, and one each in the left upper and lower lobes, Fig. 1a-f). The largest nodule (15 mm) was a mixed GGO lesion at the apical segment of the right upper lobe, and the other pure GGO lesions were 3–6 mm in size. The patient was presumptively diagnosed with bilateral SMPLC. Her preoperative staging work-up indicated no signs of lymphadenopathy or distal metastasis. Additionally, the forced expiratory volume (FEV)1 and FEV1/forced vital capacity (percentage predicted) in her preoperative pulmonary function tests were 2.38 L and 102.3, respectively.

**Fig. 2:**
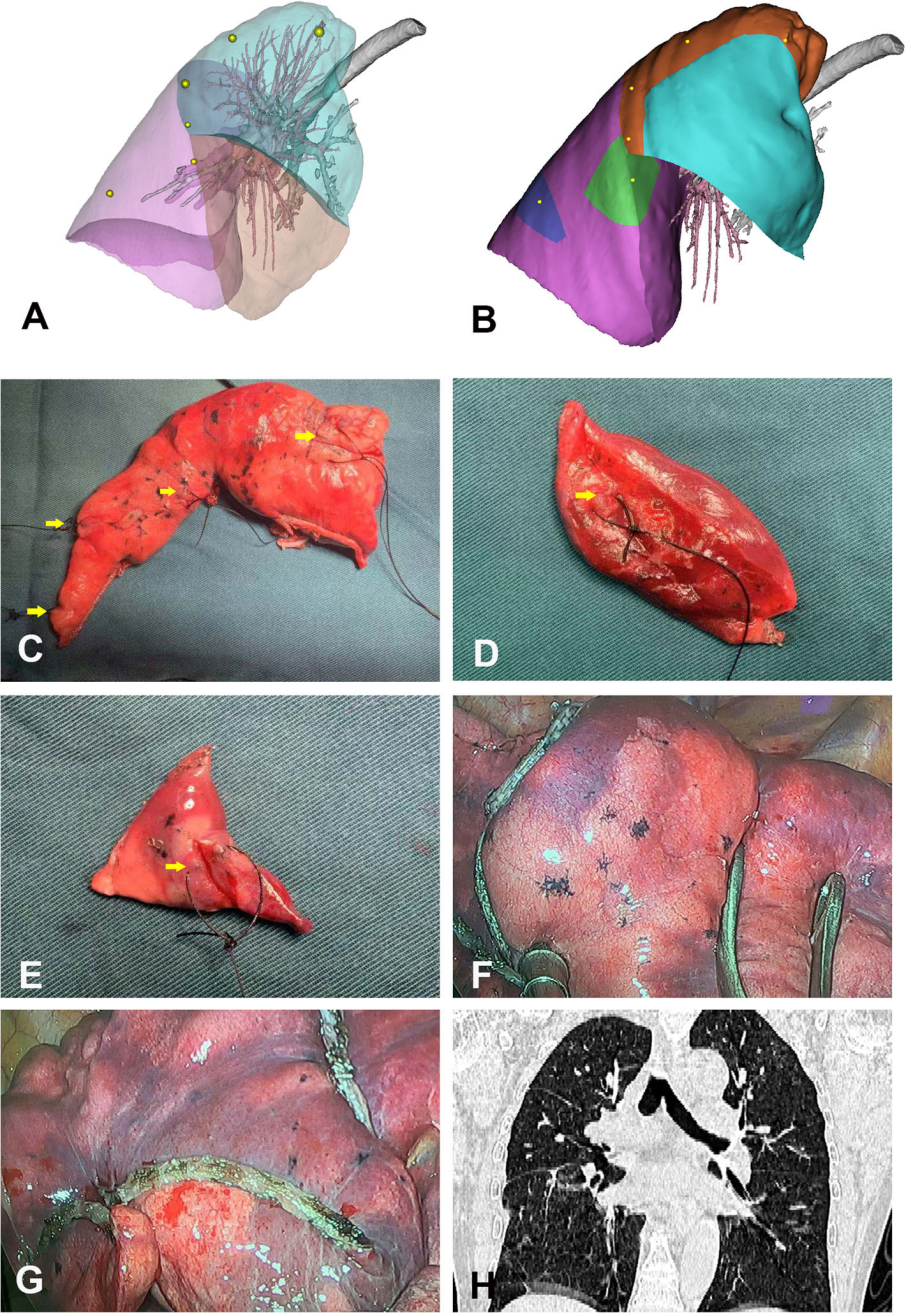
Illustration of the surgical procedure used in the patient. **a** Three-dimensional computed tomography lung reconstruction of the right lung showing the relative positions of the nodules. **b** A schematic diagram of the resection of lesions in the right lobe. **c**-**e** Resected specimens showing adequate surgical margins (arrows: C, right upper lobe; D, E, right lower lobe). **f** Intraoperative view of the remaining right upper lobe after resection. **g** Intraoperative view of the remaining right lower lobe after resection. **h** Postoperative computed tomography images showing re-expansion of the right lobe with a large lung volume

We used commercially available medical image processing software (Materialise Interactive Medical Image Control System [MIMICS], version 20.0, Materialise, Leuven, Belgium) to process the computed tomography (CT) data acquired from axial imaging and create an accurate 3D, printable model. The 3D models demonstrate each of the lung lobes and display the reconstructed lungs and lung nodules in their real relative positions. Considering the relative position of the nodules in the lobes in the 3D-CTLR model in association with the size and number of nodules, we proceeded with uniportal VATS apical segmentectomy combined with wedge resection of the posterior segment of the right upper lobe and wedge resections of the right lower lobe (Fig. 2a-g).

Pathological examination confirmed six primary lung cancers (right upper lobe: one invasive adenocarcinoma (IAC), minimally invasive adenocarcinoma (MIA), and one adenocarcinoma in situ (AIS); right lower lobe: one MIA and one AIS). Chest CT 1 month after the operation showed that the right lung had good lung retention, and the FEV1 and FEV1/forced vital capacity (percentage predicted) in her postoperative pulmonary function test were 2.22 L and 94.4, respectively.

## Discussion and conclusion

Surgical strategies for SMPLC present challenges. Each nodule’s position and size differ; therefore, an individualized approach for the design of a surgical strategy for each patient with SMPLC is required. When the main lesion is removed, if surgical resection does not seriously affect pulmonary function, as many of the nodules in the same lobe or other lobes as possible are also surgically removed [4]. Therefore, it is very important to design an optimized surgical strategy prior to the operation. Herein, we provide a typical clinical example to demonstrate the preoperative planning procedure for the use of 3D-CTLR to reconstruct lobes containing nodules, virtual simulation, and intraoperative guidance of VATS resection.

Surgical procedures for SMPLC are determined according to tumor size and location and cardiopulmonary function [5]. As pneumonectomy is associated with a high risk of postoperative respiratory failure and should be avoided whenever possible, sublobar resection for the first tumor, including wedge resection and segmentectomy under VATS, remains a good alternative; this is especially true for bilateral tumors, which usually require the sacrifice of less lung to preserve the feasibility and safety of contralateral resection [6]. There were four nodules in the right upper lobe of our patient. Without 3D-CTLR, our surgical plan may have included right upper lobectomy or apical posterior segmentectomy. We analyzed the relative positions of these nodules according to a 3D model, and we then chose sublobar resection, that is, apical segmentectomy with wedge resection of the posterior segment. This surgical strategy preserved the anterior segment and most of the posterior segment of the right upper lobe, which avoided right upper pneumonectomy. Therefore, we believe that the use of 3D-CTLR to plan the surgery, while ensuring nodular resection, can preserve lung tissue as much as possible. The latter is particularly important for patients with SMPLC. Of course, there are many factors and issues that need to be discussed, but through these this case, we wish to convey that for patients with SMPLC, preoperative 3D-CTLR can help us design a surgical strategy, that is, the extent and method of resection.

## Data Availability

The datasets used and/or analyzed during the current study are available from the corresponding author on reasonable request.

## Abbreviations

SMPLC: Synchronous multiple primary lung cancer
VATS: Video-assisted thoracoscopic surgery
3D-CTLR: Three-dimensional computed tomography lung reconstruction
